# Pre-Hypertension among Young Adults (20–30 Years) in Coastal Villages of Udupi District in Southern India: An Alarming Scenario

**DOI:** 10.1371/journal.pone.0154538

**Published:** 2016-04-29

**Authors:** Sanjay Kini, Veena G. Kamath, Muralidhar M. Kulkarni, Asha Kamath, Siddharudha Shivalli

**Affiliations:** 1 Department of Community Medicine, K. S. Hegde Medical Academy (KSHEMA), NITTE University, Deralakatte, Mangalore- 575 108, Karnataka, India; 2 Department of Community Medicine, Kasturba Medical College, Manipal University, Manipal- 576102, Karnataka, India; 3 Department of Community Medicine, Yenepoya Medical College, Yenepoya University, Mangalore-575018, Karnataka, India; Mexican Social Security Institute, MEXICO

## Abstract

**Introduction:**

According to Joint National Committee-7 (JNC-7) guidelines, a systolic blood pressure (SBP) of 120 to 139 mm Hg and/or diastolic blood pressure (DBP) of 80 to 89 mm Hg is considered as pre-hypertension. Existing evidence suggest that the cardiovascular morbidities are increasing among pre-hypertensive individuals compared to normal.

**Objective:**

To assess the magnitude and factors associated with pre-hypertension among young adults (20–30 years) in coastal villages of Udupi *Taluk* (an area of land with a city or town that serves as its administrative centre and usually a number of villages), Udupi District, Karnataka state, India.

**Design:**

Community based cross sectional study

**Setting:**

6 (out of total 14) coastal villages of Udupi *Taluk*, Karnataka state, India.

**Sample:**

1,152 young adults (age group: 20–30 years) selected by stratified random sampling in 6 coastal villages of Udupi *Taluk*, Karnataka state, India

**Method:**

A semi structured pre-tested questionnaire was used to elicit the details on socio-demographic variables, dietary habits, tobacco use, alcohol consumption, physical activity, family history of hypertension and stress levels. Anthropometric measurements and blood pressure were recorded according to standard protocols. Serum cholesterol was measured in a sub sample of the study population. Multivariate logistic regression was applied to identify the independent correlates of pre-hypertension among young adults (20–30 years).

**Main Outcome Measures:**

Prevalence, Odds ratio (OR) and adjusted (adj) OR for pre-hypertension among young adults (20–30 years).

**Results:**

The prevalence of pre-hypertension in the study population was 45.2% (95%CI: 42.4–48). Multivariate logistic regression analysis revealed that age group of 25–30 years (adj OR: 4.25, 95% CI: 2.99–6.05), white collared (adj OR: 2.29, 95% CI: 1.08–4.85) and skilled occupation (adj OR: 3.24, 95% CI: 1.64–6.42), students (adj OR: 2.46, 95% CI: 1.22–4.95), using refined cooking oil (adj OR: 0.53, 95% CI: 0.29–0.95), extra salt in meals (adj OR: 2.46, 95% CI: 1.52–3.99), salty food items (adj OR: 6.99, 95% CI: 3.63–13.48), pre-obese (adj OR: 1.66, 95% CI: 1.03–2.67) and obese (adj OR: 9.16, 95% CI: 2.54, 36.4) were the significant correlates of pre-hypertension.

**Conclusion:**

In the study population, prevalence of pre-hypertension among young adults (20–30 years) was high (45.2%). Biological (age 25–30 years, pre-obesity and obesity) and behavioral (sedentary occupation, intake of extra salt in meals/salty food and not using refined cooking oil) factors were associated with pre-hypertension. Study emphasizes the need of community based screening of pre-hypertension under National Rural Health Mission. It also provides apt information for the evidence based designing of interventions for lifestyle modifications among high risk young adults in the study area.

## Introduction

The National High Blood Pressure Education Program Coordinating Committee of the National Heart, Lung, and Blood Institute in United States released the Seventh Report of the Joint National Committee (JNC) on Prevention, Detection, Evaluation, and Treatment of High Blood Pressure (the JNC 7 report), on May 14^th^, 2003 [1]. According to this report, a systolic blood pressure (SBP) less than 120 mm Hg and diastolic blood pressure (DBP) less than 80 mm Hg was considered as normal blood pressure and a systolic BP of 120 to 139 mm Hg and/or a diastolic BP of 80 to 89 mm Hg was considered as pre-hypertension. The definition of pre-hypertension remains the same in the latest JNC-8 report published in 2014 [2]. The objective of defining this classification of blood pressure was to draw the attention of clinicians and public in the prevention of hypertension in healthy people in the pre-hypertensive range of blood pressure.

Various studies conducted in India have shown the prevalence of pre-hypertension in the age group 20–30 years to be ranging from 24.6 to 65% [3–8]. A large proportion of pre-hypertensives have at least one cardiovascular risk factor and there is a moderate to high risk of pre-hypertensives progressing to hypertension [5,9,10].

Coastal populations have higher risk of hypertension. The tradition of salting and drying fish increases the salt intake in these populations as the amount of salt used for fish conservation is high [11]. The high amount of salt used for salting fish can have an undesirable effect on blood pressure [12]

Hence, there is a great need of detecting pre-hypertension in young adults residing in coastal area. Assessing the magnitude and the factors associated with pre-hypertension would be of great use in evidence based modification in ongoing strategies.

## Objective

To assess the magnitude and factors associated with pre-hypertension among young adults (20–30 years) in coastal villages of Udupi *Taluk*, Udupi District, Karnataka state, India.

## Methods

### Ethical approval

Ethical clearance was obtained from the Institutional Ethics Committee of Kasturba Medical College, Manipal University, Manipal, Karnataka state, India (IEC 51/2012 dated 14/03/2012) and it followed the tenets of the Declaration of Helsinki. Informed written consent was taken from all the study participants for voluntary participation in local language, Kannada.

### Study setting

Udupi is a coastal district of Karnataka state, India and is bound by Arabian Sea in west and Western Ghats in the east. It has a total population of 843,300 in rural areas and 334,061 in urban areas. The striking feature of the district is the high literacy rate of 86.24%, a commendable female literacy rate of 81.58% and a favorable sex ratio of 1094 females per 1,000 males. The district is divided into 3 *taluks* (Udupi, Kundapur and Karkala). *Taluk* is an area of land with a city or town that serves as its administrative centre and usually a number of villages. The present study was conducted in Udupi *taluk* of the Udupi district. The major occupation of men folk is agriculture and fishing, while the women folk work in paddy fields, sell fish and roll beedis (cigarette filled with tobacco flake and wrapped in a leaf). Medical facilities are available within 5 to 10 km radius in the villages. Health services are provided through a network of Primary Health Centres, Community Health Centres and also District hospital. The coastal area of Udupi *taluk* consists of 14 villages with a population of 47,817 living in 8,684 families.

### Study design and sample

A community based cross-sectional study was conducted in, Udupi *taluk*, Udupi district, Karnataka, India from Oct 2011-Oct 2013. We selected 6 coastal villages for the study by simple random sampling. The total population of the young adults (20–30 years) in selected 6 villages was 9546 (males: 4566 and females: 4980).

Sample size was estimated using Epi Info^™^ 7.1.5 software. Based on the reported pre-hypertension prevalence of 25.63% (~26%) among 18–30 year age group by Bharadwaj R et al [8], a population size of 9546, a design effect of 2 and an expected response rate of 80%, the study required a sample size of 1102 for estimating the expected proportion with 4% absolute precision and 95% confidence.

#### Inclusion and exclusion criteria

Young adults in the age group of 20–30 years were included. Known hypertensive (both primary and secondary) already on treatment for hypertension, pregnant women, and those not consenting for voluntary participation were excluded from the study.

#### Sampling

Required numbers of young adults were selected by stratified sampling with proportional allocation. Stratification was based on the locality and gender. The first author went to the centre of the locality and the nearest house was taken as the first house for the study in that locality. He then moved in one particular direction and covered all the houses till he achieved the required sample for the locality. At the house, all the members satisfying the inclusion criteria were considered eligible for the study.

#### Study tool

A semi structured pre-tested questionnaire [S1 File] was administered to the subjects after obtaining their consent. The questionnaire comprised of details on socio-demographic variables, dietary habits, tobacco use, alcohol consumption, physical activity, family history of hypertension and stress levels. Anthropometric measurements and blood pressure were recorded. Total Serum Cholesterol was measured for a randomly selected sub sample of 30% population which included equal number of pre-hypertensives and normotensives. During the initial part of the study, all the individuals who were found to be pre-hypertensives and normotensives were informed about the serum total cholesterol estimation and if they consented for the test, blood sample was collected till the required sample size was achieved.

Family structure was categorized as nuclear (consisting of a couple and/or their dependent children), joint (a number of married couples and their children where all men are related by blood, who live under one roof, eat from common kitchen, hold property in common, and participate in common family worship) or three generation (grandparents, parents and children) [13]. Socio-economic status was assessed based on the modified version of *Uday Parikh* scale for rural areas in India [14]. Occupation was stratified as professional, white collared job, skilled worker, semiskilled worker, unskilled worker, housewife, student and unemployed [S2 File]. Literacy status was classified as literate (if she can read and write with understanding in any language) or illiterate (can neither read nor write or can read but cannot write in any language) and literacy level was the highest level of education completed (Census India 2011) [15].

Dietary habits: Subjects were asked whether they consume a vegetarian or mixed diet and the frequency of intake was assessed. Mutton, pork, beef were grouped as red meat. Butter and fried items were grouped as fatty food. Pickle, *papadom* (a large disc of unleavened spiced bread made from ground lentils fried in oil), dried fish, chips were grouped as salty foods. Green leafy vegetables, fruits and juice were grouped as protective foods. Frequency of intake of these were categorized as either daily (or 4–7 times per week), 2–3 times per week or occasionally (less frequently than once a week). Tea and coffee were grouped as beverages. Frequency was categorized as either once daily, twice daily or ≥ thrice daily.

Weight was measured to the nearest 100 gms, in light clothing, using a standard weighing machine after correcting the zero error. Height was measured to the nearest 0.5 cm with the person standing upright against the wall with heels together and touching the wall, and the head held in upright position. Body mass Index was computed as kilogram of body weight per meter square height (kg/m^2^).

Physical activity, smoking and alcohol consumption was recorded using standard questionnaires [16–19]. Family history of hypertension was recorded. Stress levels were measured using a perceived stress scale [20]. The following procedure was used to record the blood pressure [21]: Participant was asked to seat comfortably, with back supported, legs uncrossed, and upper arm bared. His/her arm was supported at heart level. Cuff bladder, encircling 80% or more of the arm circumference, was tied over the right arm. First, the systolic blood pressure was estimated by palpatory method and then systolic and diastolic pressures by auscultatory method. The first and last audible sounds were recorded as systolic and diastolic pressure, respectively. Measurements were given to the nearest 2 mm Hg. Two readings were taken at least 10 minutes apart and average of 2 readings was considered. Participants were classified as per the JNC-7 classification [1]. All the blood pressure measurements were recorded by the first author (SK) to avoid the inter-observer variation.

Serum cholesterol was estimated in a sub-sample of the pre-hypertensives randomly identified from the survey and an equal number of age and gender matched normotensives. Subjects were asked to be nil per oral for at least 10 hours before drawing the sample. A visit to the subject’s house was made by SK early in the morning on the day of sample collection. Three ml of blood was drawn under aseptic precautions from the subject by the investigator. It was collected in a vacutainer and was sent immediately to Biochemistry laboratory of Kasturba Medical College, Manipal. Cholesterol estimation was based on oxidase-peroxidase method by cobas^®^ 6000 analyzer series (Roche Diagnostics International Ltd, Switzerland) autoanalyser. Total cholesterol values were classified as per adult treatment panel (ATP) guidelines [22].

### Statistical analysis

Data was analyzed using Statistical Package for the Social Sciences (SPSS) for Windows, Version 16.0. Chicago, SPSS Inc. Results were expressed as frequencies and proportions for categorical variables and mean and standard deviations for continuous variables. Univariate analysis was done using chi square test and the strength of association was reported as Odds ratio (OR) with 95% confidence interval (CI). Multiple logistic regression was applied to study the independent association of risk factors and reported as adjusted OR with 95% CI. A two sided p<0.05 was considered as statistically significant.

## Results

A total of 1152 eligible young adults in the age group of 20–30 years were approached and all of them participated in the study (response rate: 100%). Table 1 shows the key socio-demographic parameters of the study population. Young adults aged from 25–30 years (n = 636, 55.2%), never married (n = 738, 64%) and those following the Hindu religion (n = 100, 86.9%) constituted more than half of study the population. Literacy rate was 98.3% (n = 1132) and joint and nuclear family systems were in vogue. As many as 63.2% (n = 728) were having moderate to heavy stress on perceived stress scale. The prevalence of pre-hypertension and hypertension among them were 45.2% (n = 521, 95% CI: 42.4–48.1%) and 3% (n = 35, 95% CI: 2.2–4.2%), respectively.

**Table 1:** Key socio-demographic parameters of young adults (20–30 years) in coastal villages of Udupi *taluk*, Karnataka, India, Oct 2013 (N = 1152)^#^.

Parameter	Male (n = 551)	Female (n = 601)	Total (N = 1152)
**Age group**			
20–24 years	255 (46.3)	261 (43.4)	516 (44.8)
25–30 years	296 (53.7)	340 (56.6)	636 (55.2)
**Marital status**			
Unmarried (Never married)	471 (85.5)	267 (44.4)	738 (64.0)
Ever married	80 (14.5)	334 (55.6)	414 (36.0)
**Religion**			
Hindu	474 (86.0)	527 (87.7)	1001 (86.9)
Muslim	52 (9.5)	58 (9.7)	110 (9.5)
Christians	25 (4.5)	16 (2.7)	41 (3.6)
**Occupation**			
Professional	12 (2.2)	10 (1.7)	22 (1.9)
White collared job	47 (8.5)	59 (9.8)	106 (9.2)
Skilled worker	158 (28.7)	48 (8.0)	206 (17.9)
Semiskilled worker	123 (22.3)	30 (5.0)	153 (13.3)
Unskilled worker	114 (20.7)	33 (5.5)	147 (12.8)
Housewife	-	273 (45.3)	273 (23.7)
Student	85 (15.4)	80 (13.3)	165 (14.3)
Unemployed	11 (2.0)	69 (11.5)	80 (6.9)
**Literacy status**			
Illiterate	10 (1.8)	10 (1.7)	20 (1.7)
Primary school	35 (6.4)	47 (7.8)	82 (7.1)
High school	213 (38.7)	244 (40.6)	457 (39.7)
Pre-university Course	162 (29.4)	99 (16.5)	261 (22.7)
Degree	122 (22.1)	185 (30.8)	307 (26.6)
Post graduate	9 (1.6)	16 (2.7)	25 (2.2)
**Type of family**			
Nuclear	272 (49.4)	269 (44.8)	541 (47.0)
Joint	224 (40.7)	256 (42.6)	480 (41.7)
Three generation	55 (10.0)	76 (12.6)	131 (11.3)
**Socioeconomic status**			
Low	67 (12.2)	35 (5.8)	102 (8.9)
Middle	463 (84.0)	541 (90.0)	1004 (87.1)
High	21 (3.8)	25 (4.2)	46 (4.0)

^**#**^figures in parenthesis indicate column wise percentages.

Univariate analysis of demographic correlates of pre-hypertension and normotension revealed that males in the age group of 25–30 years had 6.45 times higher likelihood of being pre-hypertensives as compared to females in the same age group. However, in the age group of 20–24 years the prevalence of pre-hypertension among males and females was almost equal (50%) [Table 2]. The proportion of prehypertensives was higher in the age group of 25–30 years (68.1%) as compared to the subjects in the age group of 20–24 years with a 3 times higher likelihood of being prehypertensives (OR:2.89, 95% CI:2.26–3.7). Among the prehypertensives there were a higher proportion of males (58.5%) as compared to females (OR:2.39, 95% CI:1.83–3.04). Among males, the proportion of prehypertensives in the age group of 25–30 years was significantly higher as compared to the age group of 20–24 years (OR:8.69, 95% CI:5.82–12.97, p<0.001), while in females this association was not found to be significant.

**Table 2:** Univariate analysis of various study variables among prehypertensive and normotensive young adults (20–30 years) in coastal villages of Udupi *taluk*, Karnataka, India, Oct 2013 (N = 1117).

Study variable	Total	Prehypertension (%)	Normotension (%)	Odds Ratio(95% CI)	p
**Gender**					
Male	526	305 (58)	221(42)	2.4 (1.88, 3.04)	**<0.001**
Female	591	216 (36.5)	375 (63.5)	1	
**Age group (years)**					
20–24	509	166 (32.6)	343(67.4)	1	**<0.001**
25–30	608	355 (58.4)	253 (41.6)	2.9 (2.27, 3.71)	
**Marital status**					
Never married	719	336 (64.5)	383 (64.3)	1.01(0.79,1.29)	0.936
Ever married	398	185 (35.5)	213 (35.7)	1	
**Occupation**					
Professional	21	6 (1.2)	15 (2.5)	1.08(0.37,3.16)	**<0.001**
White collared	103	49 (9.4)	54 (9.1)	**2.46(1.30,4.63)**	
Skilled	201	134 (25.7)	67 (11.2)	**5.42(3.04,9.69)**	
Semiskilled	146	68 (13.1)	78 (13.1)	**2.36(1.30,4.29)**	
Unskilled	138	76 (14.6)	62 (10.4)	**3.32(1.82,6.07)**	
Housewife	267	102 (19.6)	165 (27.7)	1.67(0.96,2.93)	
Student	163	65 (12.5)	98 (16.4)	1.80(0.99,3.24)	
Unemployed	78	21 (4.0)	57 (9.6)	1	
**Religion**					
Hindu	970	437 (83.9)	533 (89.4)	0.64(0.34,1.20)	**0.023**
Muslim	106	61 (11.7)	45 (7.6)	1.06(0.51,21.9)	
Christian	41	23 (4.4)	18 (3.0)	1	
**Literacy status**					
Illiterate	19	10 (1.9)	9 (1.5)	1.55(0.46,5.22)	0.175
Primary school	82	43 (8.3)	39 (6.5)	1.54(0.61,3.87)	
High school	441	205 (39.3)	236 (39.6)	1.21(0.52,2.79)	
Pre-university Course	250	129 (24.8)	121 (20.3)	1.49(0.63,3.48)	
Graduate	301	124 (23.8)	177 (29.7)	0.98(0.42,2.27)	
Post graduate	24	10 (4.19)	14 (2.3)	1	
**Type of family**					
Nuclear	534	228 (43.8)	296 (49.7)	0.66(0.44,0.98)	**0.081**
Joint	470	227 (43.6)	243 (40.8)	0.80(0.54,1.20)	
Three generation	123	66 (12.7)	57 (46.3)	1	
**Socioeconomic status**					
Low	99	48 (9.2)	51 (8.6)	1	**0.028**
Middle	974	444 (85.2)	530 (88.9)	0.89(0.58,1.34)	
High	44	29 (5.6)	15 (2.5)	2.05(0.98,4.29)	

Table 2 shows a very strong association between personal habits and pre-hypertension.

Among those who gave a history of smoking, 50 were current smokers and two were ex-smokers. The median duration of smoking among smokers was 4 years [Inter Quartile Range (IQR): 2, 5.5]. There was a higher proportion of smokers among the prehypertensives (7.7%) as compared to normotensives (7.7% vs. 2.0%, p<0.001), with a four times higher likelihood of prehypertensives being smokers as compared to normotensives (OR = 4.04; 95% CI: 2.1–7.80). Similar observations were made with respect to consumption of other forms of tobacco. The proportion of subjects who consumed other forms of tobacco was observed to be higher among prehypertensives (9.0%) as compared to normotensives (3.2%), (P<0.001) with an OR of 3.01 (95% CI = 1.74, 5.20). Study subjects giving a history of alcohol consumption were all current consumers. Consumption of alcohol was found to be significantly higher (15.4%) among prehypertensives as compared to normotensives (4.4%), (p<0.001) with an OR of 3.97 (2.51, 6.29). For the ever consumers the median duration of usage was 5 years [IQR = (2, 5)].

As shown in Table 3, BMI and waist circumference showed a significant association with pre-hypertension. The risk of pre-hypertension increased with increasing levels of BMI and this trend was statistically significant. The preobese and obese category was 2.24 times and 5.74 times more likely to be prehypertensive as compared to normal category. The underweight category had a protective effect on pre-hypertension. Among the normotensives mean BMI (kg/m2) was 20.63±3.15 and among prehypertensives it was 22.2±3.95. A mean difference of 1.57 (95% CI: 1.15–1.99) was found to be statistically significant. Central obesity as measured by waist circumference was significantly associated with pre-hypertension and prehypertensives had a 3.7 times more likelihood of having central obesity as compared to normotensives. Truncal obesity was present in 24% of the prehypertensives and 19.1% normotensives thus showing 1.33 times higher likelihood of truncal obesity among prehypertensives.

**Table 3:** Association between various biological and behavioural factors with prehypertension among young adults (20–30 years) in coastal villages of Udupi *taluk*, Karnataka, India, Oct 2013 (N = 1117).

Study variable	N	Pre-hypertension (%)	Normotension (%)	Odds ratio (95% CI)	p
Smoking	52	40 (7.7)	12 (2.0)	4.04 (2.10–7.80)	<0.001
Other forms of tobacco use	66	47 (9.0)	19 (3.2)	3.01 (1.74–5.20)	<0.001
Alcohol use	106	80 (15.4)	26 (4.4)	3.97 (2.51–6.29)	<0.001
Family history of hypertension	312	163 (31.3)	149 (25.0)	1.36 (1.05–1.77)	0.019
**Body Mass Index (BMI)**					
Underweight	257	86 (16.5)	171 (28.7)	0.57 (0.42–0.77)	<0.001
Normal	703	327 (62.8)	376 (63.1)	1	
Pre-obese	133	88 (16.9)	45 (7.6)	2.24 (1.52–3.31)	
Obese	24	20 (3.8)	4 (0.7)	5.74 (1.94–16.99)	
**Waist circumference (WC) and waist hip ratio (WHR)**					
Central obesity	33	25 (4.8)	8 (1.3)	3.70 (1.65–8.28)	0.001
Truncal obesity	239	125 (24.0)	114 (19.1)	1.33 (1.00–1.77)	0.048
**Stress levels**					
No or mild stress	389	86 (16.5)	303 (50.8)	1	<0.001
Moderate to heavy stress	728	435 (83.5)	293 (49.2)	5.23 (3.94–6.93)	
**Physical activity**					
Light	730	326 (62.6)	404 (67.8)	0.69(0.44–1.10)	0.151
Moderate	305	151 (29.0)	154 (25.8)	0.84 (0.51–1.38)	
Heavy	82	44 (8.4)	38 (6.4)	1	
**Cooking oil**					
Coconut oil	737	358 (68.7)	379 (63.6)	1.25 (0.98, 1.61)	0.071
Palm oil	416	195 (37.4)	221 (37.1)	1.01 (0.79, 1.29)	0.905
Pure ghee	16	11 (2.1)	5 (0.8)	2.54 (0.88, 7.38)	0.074
Refined oil	75	31 (6.0)	44 (7.4)	0.79 (0.49, 1.27)	0.340
**Dietary habits**					
Non vegetarian	1094	510 (97.9)	584 (98.0)	1.05 (0.45, 2.39)	0.908
Red meat consumption	563	270 (51.8)	293 (49.3)	1.10 (0.87, 1.39)	0.375
Extra salt in meals	112	76 (14.6)	36 (6.0)	**2.65 (1.75, 4.02)**	**< 0.001**
Salty food	1008	509 (97.7)	499 (83.7)	**8.24 (4.47, 5.21)**	**< 0.001**
Protective food	1111	516 (99.0)	595 (99.8)	0.17 (0.02, 1.48)	0.103
Egg	1082	505 (96.9)	577 (96.8)	1.05 (0.52, 2.04)	0.911
Fish	1089	509 (97.9)	580 (97.3)	1.17 (0.54, 2.49)	0.684
Beverages	1094	507 (97.3)	587 (98.3)	0.55 (0.23, 1.29)	0.167
Chicken	1063	500 (96.0)	563 (94.5)	1.39 (0.79, 2.44)	0.242

It was observed that the type of cooking oil consumed did not have any effect on pre-hypertension. The type of diet also had no bearing on the risk of pre-hypertension. Among various type of food items, consumption of salty foods and addition of extra salt in meals was significantly associated with pre-hypertension (p<0.001). It was also found that increased frequency of salty food intake was significantly associated with increased risk of pre-hypertension. A 20 times higher likelihood of being prehypertensive was observed among daily consumers of salty food (OR:20.54, 95% CI:10.52–40.09). Similarly those who consumed salty food 2–3 times per week had 8 times higher likelihood and those who consumed occasionally had 6 times higher likelihood of being prehypertensive as compared to non-consumers.

As shown in Table 4, physical activity did not show any association with pre-hypertension. Moderate to heavy stress was seen to have statistically significant association with pre-hypertension with an OR of 5.23 (95% CI:3.94–6.93). The proportion of prehypertensives with a family history of hypertension was significantly higher than normotensives (OR:1.36, 95% CI:1.05–1.77).

**Table 4:** Multivariate logistic regression analysis for the correlates of pre-hypertension among young adults (20–30 years) in coastal villages of Udupi *taluk*, Karnataka, India, Oct 2013 (N = 1152).

Correlate	Adjusted Odds ratio	95% CI
**Occupation**		
Unemployed	1	-
Professional	0.59	(0.17, 1.95)
White collared	**2.29**	**(1.08, 4.85)**
Skilled	**3.24**	**(1.64, 6.42)**
Semiskilled	1.37	(0.68, 2.75)
Unskilled	1.82	(0.89, 3.75)
Housewife	0.67	(0.34, 1.31)
Student	**2.46**	**(1.22, 4.95)**
**Religion**		
Christians	1	-
Hindu	0.52	(0.23, 1.14)
Muslim	1.38	(0.55,3.42)
**Cooking oil**		
Refined oil	**0.53**	**(0.29, 0.95)**
**Extra salt in meals**		
Yes	**2.46**	**(1.52, 3.99)**
**Age group**		
25–30 years	**4.25**	**(2.99, 6.05)**
**Salty food**		
Yes	**6.99**	**(3.63, 13.48)**
**Stress category**		
No stress	1	-
Mild	0.13	(0.05, 0.37)
Moderate	0.77	(0.29, 2.07)
Severe	1.98	(0.38, 12.84)
**Body Mass Index**		
Normal	1	-
Underweight	0.78	(0.54, 1.12)
Pre-obese	**1.66**	**(1.03, 2.67)**
Obese	**9.16**	**(2.54, 36.40)**

It was observed that the borderline total cholesterol category (200–240 mg/dl) comprised a higher proportion of prehypertensives as compared to normotensives and the borderline category had twice the likelihood of being prehypertensives as compared to normal levels of cholesterol (OR:2.24, 95% CI:1.3–3.85). Even the high total cholesterol (>240mg/dl) category showed a higher risk of pre-hypertension but results were not statistically significant. The mean levels of total cholesterol (mg/dl) were higher among prehypertensives 183.34±31.20, as compared to normotensives who had a mean of 174.41±29.44 (p = 0.06). The mean difference was found to be 8.93 (95% CI: 2.5–15.37).

Multivariate logistic regression analysis revealed that age group of 25–30 years, white collared and skilled occupation, students, refined cooking oil, extra salt in meals, salty food items, preobese and obese were significant correlates of pre-hypertension.

## Discussion

### Main findings

In the study population, prevalence of pre-hypertension among young adults (20–30 years) was high (45.2%; 95% CI: 42.4–48.1%). Biological (age group of 25–30 years, pre-obesity and obesity) and behavioral (sedentary occupation, intake of extra salt in meals and salty food) factors were associated with pre-hypertension.

### Interpretation

The prevalence of pre-hypertension in the study population was 45.2% (95% CI = 42.4, 48.0). The results are consistent with a study done by Vimala et al [23] in Kerala where the prevalence of pre-hypertension was 52.52% in the age group of 18–30 years. Since the life style factors like dietary habits and socio-demographic factors are almost similar in coastal Karnataka and Kerala, the proximity of the results can be justified.

Similar to the present study, most of the studies have reported a higher prevalence of pre-hypertension among males than females. Mungreiphy NK et al [6] conducted a study among 360 young adults in the age group of 20 to 30 years in three different places in India (Delhi, Manipur and Kerala) and found the prevalence of pre-hypertension among males to be higher at around 54% and in females at around 10%. In the present study the risk of pre-hypertension increased with increasing levels of BMI and this trend was statistically significant. Results are similar to a study done by Singh RB et al [24] wherein they found out the mean BMI (kg/m^2^) among pre-hypertensive women as 22.6 ± 2.3 and normotensive women as 20.1 ± 2.5. Among men the mean BMI was 22.6 ± 3.1 and among normotensive men it was 20.2 ± 2.5.

In the present study the type of diet (veg vs. non-veg) had no bearing on pre-hypertension whereas, 20 times higher likelihood of being prehypertensive was observed among daily consumers of salty food (OR: 20.54, 95% CI:10.52–40.1). Ray S et al [25] in their study found a significant difference in the consumption of non-vegetarian foods among prehypertensives (25.7%) and normotensives (4.7%), p<0.001. It was also found that proportion of subjects consuming extra salt in meals, consuming salty food like pickle, consuming ghee/butter were significantly higher among prehypertensives (33%, 31.7% and 59.1%) as compared to normotensives (7.2%, 10.2% and 14.1%). In the present study, the proportion of prehypertensives with a family history of hypertension was significantly higher than normotensives (OR:1.36, 95% CI:1.05–1.77). A study conducted by Al-Majed et al [26] revealed that the proportion of subjects with a family history of hypertension was significantly higher among prehypertensives (42.6%) as compared to normotensives (40.0%).

In the present study on multivariate logistic regression analysis age group of 25–30 years, white collared and skilled occupation, students, refined cooking oil, extra salt in meals, salty food items, pre-obese and obese were found to be significant correlates of pre-hypertension. Gupta et al [7] in their study found that on multivariate logistic regression analysis high dietary fat (20 g/day), low fruits and vegetables intake, overweight/obesity, central obesity, high cholesterol, Metabolic syndrome (ATP-III defined) as significant risk factors for pre-hypertension.

To address the rising burden non-communicable diseases, government of India has launched National Programme for Prevention and Control of Diabetes, Cardiovascular Diseases and Stroke (NPDCS) in 2008 on pilot basis [27]. It envisages ‘opportunistic’ community based screening for hypertension among >30 years old at grass root level by auxiliary nurse midwife. However, in the coastal populace of Udupi district, screening should begin at the age of 20 years to identify the high risk group and plan apt interventions.

## Limitation

The dietary intake was measured only in terms of frequency and not the quantity. The serum cholesterol estimation was done only in a sub sample of study population because of lack of funds. Associations between study variables may not imply causality owing to cross-sectional study design.

## Conclusion

The prevalence of pre-hypertension in young adults was 45.2%. The risk factors found to be strongly associated with pre-hypertension were age group of 25–30 years, intake of extra salt in meals, salty food intake, pre-obesity, and obesity. Screening strategies for pre-hypertension should be initiated at an early age in the community. The importance of lifestyle modifications with respect to personal habits, dietary habits, physical exercise, and relaxation techniques needs to be impressed upon young adults in the community.

## Supporting Information

S1 File Questionnaire in English. (DOC)

S2 File Classification of occupation. (DOC)

S3 File STROBE checklist. (DOCX)
