# Supplementary material for: Pre-Hypertension among Young Adults (20–30 Years) in Coastal Villages of Udupi District in Southern India: An Alarming Scenario
Source: PLoS One. 2016 Apr 29;11(4):e0154538. doi: 10.1371/journal.pone.0154538 (PMC4851369; doi:10.1371/journal.pone.0154538)
Supplement: S1 File — (DOC) [file pone.0154538.s001.doc]

**Questionnaire**

Date of Interview:

1. **Identification:**

1. Age:_____ yrs
2. Sex: Male/female
3. Marital status: Unmarried/Married/Widowed/Widower/Divorced/Separated
4. Occupation:
5. Religion: Hindu/Muslim/Christian/Others
6. Literacy status: Illiterate/Primary school/High school/PUC/Degree/Post-Graduate
7. Address:
8. Type of family: Nuclear/ Joint/ 3 generation
9. Contact no:
10. **Socio-economic status: Annexure (Modified Uday Parikh socio-economic status scale)**
11. **Diet**:
12. What type of diet do you take? Veg__. Mixed___.
13. If non veg. Mention whether you consume the following items

|  | Yes | No | If yes then frequency |
| --- | --- | --- | --- |
| Egg |  |  |  |
| Fish |  |  |  |
| Chicken |  |  |  |
| Mutton |  |  |  |
| Pork |  |  |  |
| Beef |  |  |  |

1. Which oil do you use for cooking? coconut oil/palm oil /pure ghee/refined oil/any others (specify)_____
2. Do you consume extra salt in your daily meals? Yes/No
3. Please indicate if you consume the following food items

| Food items | Yes | No | If yes then frequency |
| --- | --- | --- | --- |
| Tea |  |  |  |
| coffee |  |  |  |
| Butter |  |  |  |
| Fried items |  |  |  |
| Pickles |  |  |  |
| Papad |  |  |  |
| Dried fish |  |  |  |
| Chips |  |  |  |
| Fruits |  |  |  |
| Green leafy vegetables |  |  |  |
| Juice |  |  |  |

1. **Personal History:**

1. **Smoking:** yes / no

If yes, then

1. Current smoker

| Type of smoking | Yes/No | Age started | Duration | Freq (no. Per day) |
| --- | --- | --- | --- | --- |
| Filter-cigarette |  |  |  |  |
| Non-filter cigarette |  |  |  |  |
| Beedi |  |  |  |  |
| Others (specify) |  |  |  |  |

1. Ex-smoker

| Type of smoking | Yes/No | Age started | Age stopped | Duration | Freq (no. Per day) |
| --- | --- | --- | --- | --- | --- |
| Filter-cigarette |  |  |  |  |  |
| Non-filter cigarette |  |  |  |  |  |
| Beedi |  |  |  |  |  |
| Others(specify) |  |  |  |  |  |

**B) Consumer of other forms of Tobacco:** yes / no

If yes, then

1 .Current consumer:

| Type of tobacco | Yes/No | Age started | Duration | Freq (no. Per day) |
| --- | --- | --- | --- | --- |
| Pan masala |  |  |  |  |
| Ghutka |  |  |  |  |
| Snuff |  |  |  |  |
| Others (specify) |  |  |  |  |

2 . Ex-consumer:

| Type of tobacco | Yes/No | Age started | Age stopped | Duration | Freq (no. Per day) |
| --- | --- | --- | --- | --- | --- |
| Pan masala |  |  |  |  |  |
| Ghutka |  |  |  |  |  |
| Snuff |  |  |  |  |  |
| Others(specify) |  |  |  |  |  |

**C) Consumer of Alcohol:** Yes / no

If yes, then

1. Current consumer:

| Type of Drink | Yes/No | Age started | Duration | Amount |
| --- | --- | --- | --- | --- |
| Country liquor |  |  |  |  |
| IMFL* |  |  |  |  |
| Beer |  |  |  |  |
| others |  |  |  |  |

1. Ex-consumer:

| Type of Drink | Yes/No | Age started | Age stopped | Duration | Amount |
| --- | --- | --- | --- | --- | --- |
| Country liquor |  |  |  |  |  |
| IMFL* |  |  |  |  |  |
| Beer |  |  |  |  |  |
| others |  |  |  |  |  |

*IMFL= Indian Manufactured foreign Liquor (whisky, brandy, rum, vodka)

1. **Physical Activity status:**

| Does your work involve mostly sitting/ standing, walking | Yes | |  | Minimum score=1 |
| --- | --- | --- | --- | --- |
| Does your work involve moderate activity, like brisk walking, fetching water from wells, moderate agricultural work like sowing seeds, weeding, painting buildings, house hold works etc | Duration /day | Score | Days/wk  (multiply by no. of days)  Eg. 4x7 | Maximum score |
|  | 45mins  >45mins-4hrs  >4-8hrs | 2  3  4 |  | 28 |
| Does your work involve vigorous manual activity | 15min-1 hr  >1-4 hrs  >4-10 hrs | 5  6  7 | 7x7 | 49 |
| Additional activities: Do you use a cycle or engage in sports? | 45mins  >45mins-4 hrs  > 4-6 hrs | 1  2  3 | 3x7 | 21 |
| Total | | | | 70 |

1. **Family History**
2. Does anyone in your family has hypertension? Yes/no. If yes, specify.

Father/mother/brother/sister/uncles/aunt/1^st^ cousins/grand parents.

1. **Vitals:**
2. Pulse rate:
3. Blood pressure: 1^st^  reading:

2^nd^ reading:

1. **Anthropometry:**
2. Height:_____cms.
3. Weight:_____kgs.
4. BMI:_______Kg/m^2^
5. Waist circumference:_____cms.
6. Hip circumference:______cms.
7. Waist-Hip ratio: ________
8. Total Cholesterol:________ mg/dl
