# Supplementary material for: Pre-Hypertension among Young Adults (20–30 Years) in Coastal Villages of Udupi District in Southern India: An Alarming Scenario
Source: PLoS One. 2016 Apr 29;11(4):e0154538. doi: 10.1371/journal.pone.0154538 (PMC4851369; doi:10.1371/journal.pone.0154538)
Supplement: S2 File — (DOC) [file pone.0154538.s002.doc]

OCCUPATION CLASSIFICATION

1. White collared job

M. D. Of big company

Proprietor of big business (whole sale) Doctor

Contractor (big buildings) Lawyer

Bank manager Engineer

Govt. Officer class I and II Professor

Company secretary (public or private sector) Chartered accountant

Postgraduate lecturer (M.A., M.Sc, M.Com….) Research scientist

2. PROFESSIONAL

Nurse School teacher

Typist, steno Policeman

Office manager Office accountant

LIC agent Pharmacist

Lab. Technician Clerk

Social worker

3. SKILLED WORKER

Mechanic Draftsman

Carpenter Blacksmith

Plumber Tailor

Electrician Surveyor

Potter Mason

Driver Weaver

Welder Photographer

Goldsmith

4. SEMI-SKILLED WORKER

Beedi roller Postman

Conductor Farmer

Small contractor Barber

Fisherman

5. UNSKILLED WORKER

Pan Shop Vegetable Seller

Fish Seller Watchman

Agricultural Labourer Dock Worker

Cleaner Attender

Dhobi Coolie

6. HOUSEWIFE

7. STUDENT

8. UNEMPLOYED
